# Eliminating Rabies in Estonia

**DOI:** 10.1371/journal.pntd.0001535

**Published:** 2012-02-28

**Authors:** Florence Cliquet, Emmanuelle Robardet, Kylli Must, Marjana Laine, Katrin Peik, Evelyne Picard-Meyer, Anne-Laure Guiot, Enel Niin

**Affiliations:** 1 Nancy OIE/WHO/EU Laboratory for Rabies and Wildlife, French Agency for Food, Environmental and Occupational Health and Safety, Malzéville, France; 2 Veterinary and Food Laboratory, Tartu, Estonia; 3 Luuvaniementie 6A-32, Helsinki, Finland; 4 Conseils en Pharmacie et Biologie, Sainte Foy les Lyon, France; 5 Veterinary and Food Board of Estonia, Tallinn, Estonia; Yale University, United States of America

## Abstract

The compulsory vaccination of pets, the recommended vaccination of farm animals in grazing areas and the extermination of stray animals did not succeed in eliminating rabies in Estonia because the virus was maintained in two main wildlife reservoirs, foxes and raccoon dogs. These two species became a priority target therefore in order to control rabies. Supported by the European Community, successive oral vaccination (OV) campaigns were conducted twice a year using Rabigen® SAG2 baits, beginning in autumn 2005 in North Estonia. They were then extended to the whole territory from spring 2006. Following the vaccination campaigns, the incidence of rabies cases dramatically decreased, with 266 cases in 2005, 114 in 2006, four in 2007 and three in 2008. Since March 2008, no rabies cases have been detected in Estonia other than three cases reported in summer 2009 and one case in January 2011, all in areas close to the South-Eastern border with Russia. The bait uptake was satisfactory, with tetracycline positivity rates ranging from 85% to 93% in foxes and from 82% to 88% in raccoon dogs. Immunisation rates evaluated by ELISA ranged from 34% to 55% in foxes and from 38% to 55% in raccoon dogs. The rabies situation in Estonia was compared to that of the other two Baltic States, Latvia and Lithuania. Despite regular OV campaigns conducted throughout their territory since 2006, and an improvement in the epidemiological situation, rabies has still not been eradicated in these countries. An analysis of the number of baits distributed and the funding allocated by the European Commission showed that the strategy for rabies control is more cost-effective in Estonia than in Latvia and Lithuania.

## Author Summary

This paper reports the strategy of oral rabies vaccination of wildlife in Estonia, the measures undertaken to check the method's efficacy and the results obtained. Initiated in autumn 2005, oral vaccination programmes resulted in a dramatic decrease in rabies incidence. All the recommended tests were regularly applied, including the systematic testing of vaccine baits prior to release in the field, serological testing and bait uptake assessment in adult and young animals as well as the typing of all rabies virus isolates. The disease was completely controlled by March 2008, with only three cases reported in summer 2009 and one case in January 2011 in areas very close to the South-Eastern border. The costs associated with rabies control have been calculated and compared on a similar basis for the three Baltic countries. The example of rabies control in Estonia shows that rabies can be quickly and successfully eliminated through successive oral vaccination campaigns by strictly following the recommendations of international organisations. These recommendations concern general strategy, vaccination method and choice of vaccine. To our knowledge, this is the first study showing extensive data from a rabies control programme. The underlying strategy, leading to rabies elimination, is advantageous in terms of cost/effectiveness.

## Introduction

Rabies has been a serious public and animal health issue in Estonia for centuries. Up to 1959, rabies was mainly urban. With the extermination of stray dogs and the compulsory vaccination of pets from 1953, Estonia was rabies free from 1960 to 1967. Sylvatic rabies spread throughout Estonia (including islands) from 1968, there being two main wildlife reservoirs: raccoon dogs (*Nyctereutes procyonoides*) and red foxes (*Vulpes vulpes*). By 2002, raccoon dogs had become the major rabies-infected wildlife species in Estonia [1].

From 1947 to 1955, one to eight people died of rabies every year [2]. No human case was registered from 1955 until 1983. The three most recent cases occurred between 1984 and 1986 (one case per year) and were caused by wild infected animals (foxes and roe deer).

The compulsory vaccination of pets and the recommended vaccination of farm animals in grazing areas did not succeed in fully eliminating rabies in Estonia because the virus was maintained in the wild fauna, especially foxes and raccoon dogs.

In May 2004, Estonia joined the European Union (EU) and could then benefit from the financial support of the European Commission for wildlife rabies control.

The natural double mutant SAG2 (avirulent Gif Street Alabama Dufferin strain)—a modified live avirulent rabies virus—was selected for the Estonian wildlife vaccination campaign. The SAG2 strain has been shown to be an effective immunogen when administered orally to red foxes. It contributed to the elimination of rabies in Switzerland [3] and France [4] and has been used in Italy since 2009 with successful results [5]. SAG2 is one of two vaccines recommended by the WHO Expert Consultation on Rabies for oral immunisation of wildlife and dogs [6].

An initial pilot vaccination trial conducted on Vormsi island (92 km^2^) in spring and autumn 2004 demonstrated the feasibility of orally vaccinating wildlife in Estonia [7]. Three vaccination campaigns with SAG2 baits were conducted in autumn 2005 in North Estonia, and in spring and autumn 2006 throughout the territory. Very encouraging results were achieved in terms of rabies incidence, bait uptake and immunisation in foxes and raccoon dogs [1].

The objective of this study was to assess the rabies situation after oral vaccination (OV) of wildlife from autumn 2005 until the end of 2010 and evaluate the financial aspects. Oral vaccination efficacy was assessed through the incidence of rabies in the country, the proportion of the fox and raccoon dog populations which consumed the bait (revealed through a tetracycline biomarker), and the rabies immunisation rates [6]. The overall cost of the OV campaign in Estonia was reported and compared to that of neighbouring countries.

## Materials and Methods

### Vaccine

The SAG2 vaccine (RABIGEN®, Virbac Laboratories, Carros, France) is a modified live attenuated rabies virus vaccine registered in the 27 countries of the EU (European Medicines Agency registration) for oral administration in baits to foxes and raccoon dogs [8]. The SAG2 virus strain was selected from SAD Bern—a sub-clone of a virus isolated from the salivary glands of a rabid dog in 1935—in a two-step process of amino acid mutation [9]. Tetracycline (150 mg per bait) was used as a biological marker to assess bait consumption.

Baits were sent from France to Estonia in refrigerated lorries (−20°C) and stored at −20°C prior to use. Before each vaccination campaign, ten baits from each batch of vaccine to be used (except in 2006 when three baits per batch were titrated) were sent to the European Union Reference Laboratory (EU-RL) in Nancy for titration as previously recommended [10], [11].

All vaccine batches from 2006 on (82 batches) have been titrated. The mean vaccine titre of the different batches used for each vaccination campaign ranged from 10^7.4^ to 10^8.9^ TCID50/dose.

### Vaccination strategy

The Republic of Estonia covers 45,227 km^2^, including ∼25,000 km^2^ of forests. Estonia is divided into 15 administrative counties, two represented by the islands of Saaremaa (2,673 km^2^) and Hiiumaa (1,023 km^2^). The country is bordered by Latvia to the South (339 km), Russia to the East (343 km), the Baltic Sea to the West and the Gulf of Finland to the North.

Wild animals were not vaccinated against rabies in Estonia until 2005, except on Vormsi island (92 km^2^), where a small-scale OV was carried out in 2004 with a manual distribution of baits. In autumn 2005, the first large-scale oral vaccination campaign of wildlife was conducted in the Northern part of Estonia (25,540 km^2^) from the Western to the Eastern border, including Estonia's islands. The vaccination area was bordered by a continuous line formed by roads, the shoreline of lake Peipsi and the river Narva. Around 0.5 million baits were spread throughout the vaccination area at a density of 20 baits/km^2^.

From 2006 to 2010 an OV programme co-financed by the EU and the Estonian state budget was implemented throughout the Estonian territory (42,914 km^2^ after exclusion of marshlands and urban areas). Both the vaccine (SAG2) and the distribution protocols were similar for the different campaigns. OV was carried out twice a year, in spring (May, early June) and autumn (September, October). Approximately 860,000 baits were dropped during each campaign at a density of 20 baits/km^2^ using small Cessna 127 fixed-wing aircraft. The baits were distributed manually by trained personnel through special tube systems in the plane. Estonia was divided into vaccination areas covering 240 km^2^ on average. Vaccines were dropped along parallel flight paths 600 m apart. Flights took place at an altitude of 100–150 m at an average speed of 160–180 km/h. No baits were dropped over urban areas, roads, lakes, rivers, deep swamps and active domestic animal pastures. A GPS system (Garmin 196) was used for navigation and to record flight data. During the campaigns, vaccines were stored at the airport in refrigerated lorries at −20°C.

A public awareness campaign was initiated at the same time as the oral vaccination programme by TV, radio and newspapers.

### Verification of vaccination efficacy

#### Passive and active surveillance: rabies incidence

Rabies has been a notifiable disease in Estonia since 1950. All suspected cases must be notified to veterinarians and relevant samples collected and submitted to the Tallinn department of the Veterinary and Food Laboratory (VFL) or to the central laboratory in Tartu. The rabies virus was detected in the brain of suspect animals and those found dead using the WHO recommended fluorescent antibody test (FAT) [12]. To confirm or rule out the disease on FAT-negative samples in the event of known contact with humans or unvaccinated animals, PCR tests were run and the virus isolated by cell culture. Brain samples from target animals collected by hunters for OV monitoring were also tested for rabies. The active surveillance in the two target species (fox and raccoon dog) was implemented through the analysis of the brain from eight animals per km^2^ between August 2008 and April 2009, then from four animals per km^2^ since August 2009, according to the recommendations of the WHO for monitoring the efficacy of OV programmes.

Both positive and negative cases were notified to the Veterinary and Food Board (VFB).

#### Bait uptake

From 2005 to 2010, jaw samples from eight animals (foxes and raccoon dogs) per 100 km^2^ of vaccinated area were collected annually by the Estonian Hunters' Society between late July of the year of OV to late March of the following year. From July 2011 on, this sample size was reduced to four animals per 100 km^2^, as previously recommended [6], [13].

A canine tooth with some alveolar bone tissue was isolated from each lower jaw. Samples of tooth and surrounding alveolar bone were tested at the VFL by fluorescence to detect tetracycline deposits, which appeared pale yellow on a blue background [14]. The age of all the animals tested was determined on the basis of a histological dental examination (cub <12 months of age, adult ≥12 months) [15].

#### Humoral response

From 2006 to 2010, at least four blood samples per km^2^ were collected from hunted animals in all Estonian counties between late July of the year of OV to late March of the following year.

Rabies antibodies were titrated using an indirect ELISA kit (Platelia Rabies II kit, Biorad, France) as previously described [1], [16]. An ELISA test was selected because it was more adapted than a seroneutralization test for samples collected in the field, which were most often of poor quality (hemolyzed, contaminated…). As the Platelia Rabies II kit was validated for use on raccoons in the USA [unpublished data], it was assumed that this validation (diagnostic specificity: 100%, diagnostic sensitivity: 79%) can be applied to raccoon dog samples. The antibody titre was calculated by comparing the sample's optical density to the standard curve obtained with the OIE reference serum of canine origin (EU-RL Nancy, France). A threshold positivity of 0.5 EU/ml was adopted.

### Phylogenetic analysis

All rabies cases detected in the vaccinated areas since the beginning of OV in 2005 were sequenced at the EU-RL in Nancy, including the latest case in January 2011.

A cohort of 48 samples from domestic and wild animals found to be FAT positive by the VFL was collected for typing. For the extraction of RNA and hnRT-PCR, 10% (w/v) brain material suspensions were prepared using DMEM medium containing antibiotics and 50% heat inactivated foetal calf serum and centrifuged at 1,500 g for 10 minutes. Viral RNA was extracted from 150 µL eluate using a Qiagen Viral RNA mini kit according to the manufacturer's instructions. First and second round polymerase chain reactions were carried out as previously described [17] giving an amplified product of 589 bp. Host RNA control (18S rRNA, 324 bp) was amplified for each sample using hnRT-PCR as previously described [18].

To verify RNA integrity and validate each negative RT-PCR result, 18S rRNA was amplified for each sample. All the samples were therefore analysed twice: once for host rRNA (18S rRNA) and the second with lyssavirus universal primers [17]. The PCR products (589-bp) were separated by electrophoresis in a 2% agarose gel and purified with a commercial kit (Nucleopsin Extract II columns, Macherey Nagel, France) according to the manufacturer's instructions. Gel purified PCR products were sequenced in both directions by Beckman Coulters Genomics (Takeley, United Kingdom) with the same specific primers used for the nested PCR amplification. The sequences obtained were assembled and edited with Vector NTI1.01 software (Invitrogen, France). After the alignment of sequenced amplified PCR products, 25 identical sequences showing 100% nucleotide identity for N gene (−400 nt) were removed from the phylogenetic analysis.

A phylogenetic tree of classical rabies virus nucleoprotein sequences was constructed using the Neighbour Joining method (p-distance model) with Mega version 5 [19]. A phylogenetic tree was established between 18 N sequences (−400 nt) from Estonia, 20 Eurasian reference sequences [Estonia (n = 1), Latvia (n = 4), Lithuania (n = 4) and Russia (n = 11)], two laboratory strains and two sequences acting as outgroup (Table 1). All the sequences reported in Estonia have been submitted to GenBank. Table 1 summarises sequence findings, detailing the year of isolation, the host species and the geographical origin of all isolates included in the phylogenetic study. The bootstrap probabilities of each node were calculated using 1,000 replicates to assess the robustness of the Neighbour Joining method. Bootstrap values over 70% were regarded as significant for phylogenetic analysis.

**Table 1:** Characteristics of rabies virus isolates included in the phylogenetic analysis.

Country	Year of isolation	Species	Isolate	Virus species	Phylogroup (N)	Source reference	GenBank accession Number
Estonia	1994	Raccoon dog	Est-RV9342	1	NEE	Bourhy et al. 1999	U43432
	2004	Raccoon dog	Est-RV0204MT720	1	NEE	This study	JN109225
	2004	Raccoon dog	Est-RV0304MT745	1	NEE	This study	JN109207
	2004	Raccoon dog	Est-RV0704MT740	1	NEE	This study	JN109218
	2004	Raccoon dog	Est-RV0804MT7498	1	NEE	This study	JN109219
	2004	Raccoon dog	Est-RV0904MT724	1	NEE	This study	JN109226
	2006	Raccoon dog	Est-TR0601060MT43	1	NEE	This study	JN109222
	2006	Raccoon dog	Est-TR0600543MT30	1	NEE	This study	JN109221
	2006	Red fox	Est-TR0603044MT90	1	NEE	This study	JN109227
	2006	Raccoon dog	Est-TR0601851MT66	1	NEE	This study	JN109220
	2006	Raccoon dog	Est-TR0601755	1	NEE	This study	JN109229
	2006	Badger	Est-TR0607377	1	NEE	This study	JN109230
	2006	Raccoon dog	Est-TR0608902	1	NEE	This study	JN109233
	2006	Raccoon dog	Est-BMT4	1	NEE	This study	JN109223
	2006	Raccoon dog	Est-TR0611366	1	NEE	This study	JN109231
	2006	Red fox	Est-TR0612855	1	NEE	This study	JN109232
	2006	Raccoon dog	Est-TR0616726	1	NEE	This study	JN109228
	2007	Cattle	Est-TA0705424MT42	1	NEE	This study	JN109224
	2011	Raccoon dog	Est-RV2011-DR0359	1	C	This study	JQ277471
Latvia	1999	Red fox	12LAT	1	NEE	Vanaga et al., 2003	AY277574
	1999	Dog	18LAT	1	NEE	Vanaga et al., 2003	AY277576
	1999	Red fox	20LAT	1	NEE	Vanaga et al., 2003	AY277577
	1999	Badger	22LAT	1	NEE	Vanaga et al., 2003	AY277578
Lithuania	2006	Raccoon dog	06LT4	1	NEE	Zienus et al., 2009	EU616717
	2006	Dog	06LT5	1	NEE	Zienus et al., 2009	EU616718
	2007	Red fox	07LT12	1	NEE	Zienus et al., 2009	EU616720
	2007	Raccoon dog	07LT14	1	NEE	Zienus et al., 2009	EU616721
Russia	/	Human	RV245RUS	1	E	Kuzmin et al., 2004	AY352475
	/	Raccoon dog	RV309 RUS	1	E	Kuzmin et al., 2004	AY352504
		Red fox	Rv1596 RUS	1	E	Kuzmin et al., 2004	AY352474
		Red fox	RV247 RUS	1	D	Kuzmin et al., 2004	AY352511
		Red fox	RV299 RUS	1	D	Kuzmin et al., 2004	AY352479
		Human	RV239 RUS	1	D	Kuzmin et al., 2004	AY352509
	/	Red fox	2072f RUS	1	C	Kuzmin et al., 2004	AY352485
	/	Human	RVHN RUS	1	C	Kuzmin et al., 2004	AY352463
	/	Red fox	409f RUS	1	C	Kuzmin et al., 2004	AY352489
	/	Red fox	3502f RUS	1	C	Kuzmin et al., 2004	AY352455
	/	Red fox	3605f RUS	1	C	Kuzmin et al., 2004	AY352467
Laboratory strains		SAG2		1		Geue et al, 2008	EF206719
		CVS		1		Tordo et al, 1986	D42112
India			Outgroup			Jayakumar et al., 2004	AF374721
China			Outgroup	1		Li et al. 1999	EU159392

(NEE = North Eastern Europe, D = centre of the European part of Russia, E = North-Western part of Russia).

### Cost of oral vaccination

The costs of OV in Estonia were evaluated from 2005 to 2010 and detailed according to bait vaccine purchases and their aerial distribution, collection of samples, laboratory tests, awareness campaigns and sundry other costs, such as investigations on suspected animals.

We also wished to compare the cost of OV in Estonia, Lithuania and Latvia. Data have been collected since 2006, when OV was first conducted throughout the territories of each of the Baltic States.

The common basis for comparison was the European Commission's annual financial contribution to the rabies control plans, although the comparison included the number of baits distributed and the vaccine strains used (when published). Data are available at http://ec.europa.eu/food/animal/diseases/eradication/legisl_en.htm. The contaminated and uninfected areas in km^2^ were evaluated on an annual basis for each country by defining a circle with a 50-km radius around each positive case [10] using mapping software. For each country, the annual data—total uninfected area of year n+1 subtracted from the total uninfected area of year n—were cumulated over the 2006–2010 period. These data assess the area freed from rabies.

Different ratios were calculated i.e. cumulated number of baits distributed over the 2006–2011 period/vaccinated area in the country, cumulated EC funding over the 2006–2010 period/area freed from rabies in the country (which are areas newly uninfected).

## Results

### Epidemiological situation before the first vaccination campaign

The epidemiological situation before the first vaccination campaign in autumn 2005 was reviewed [1].

Briefly, from 1994 to 2005, the number of rabies cases ranged from 74 cases in 1995 to 814 in 2003 (Table 2). The distribution of cases among species has been relatively stable over the years. During the 1968–2005 period, most cases involved wildlife (71–76% of all rabies cases), whereas farm animals accounted for 6%, dogs and cats for 18–23%. From 1968 to 2001, red foxes were the most frequently infected animals, but the number of infected raccoon dogs progressively increased over this same period. Since 2002, the raccoon dog has thus become the main reservoir (47.4% of rabies cases in 2005). Other wildlife, such as badgers, deer, rabbits, hedgehogs, ferrets, squirrels, lynx, minks, weasels, hares, marten and mice, have no epidemiological role in rabies transmission (3% of rabies cases in 2005).

**Table 2:** Number of rabies cases from 2000 to May 2011 in the main species in Estonia.

Year	Dog	Cat	Cattle	Equine	Other domestic	Total Domestic animals	Fox	Raccoon dog	Other wildlife	Total Wildlife	Total
2000	11	4	19	0	2	***36***	64	26	3	***93***	**129**
2001	6	12	11	1	0	***30***	74	60	3	***137***	**167**
2002	24	22	20	1	0	***67***	153	193	9	***355***	**422**
2003	34	28	51	1	3	***117***	316	361	20	***697***	**814**
2004	24	20	15	0	1	***60***	92	151	11	***254***	**314**
2005	6	8	19	3	1	***37***	95	126	8	***229***	**266**
2006	5	4	3	1	0	***13***	38	60	3	***101***	**114**
2007	0	0	2	0	0	***2***	0	1	1	***2***	**4**
2008	1	0	0	0	1	***2***	1	0	0	***1***	**3**
2009	0	0	0	0	0	***0***	3	0	0	***3***	**3**
2010	0	0	0	0	0	***0***	0	0	0	***0***	**0**
2011*	0	0	0	0	0	***0***	0	1	0	***0***	**1**
**Total**	**111**	**98**	**140**	**7**		**364**	**836**	**979**		**1872**	**2237**
**%**	**5.0**	**4.4**	**6.3**	**0.3**		**16.3**	**37.4**	**43.7**		**83.7**	**100**

*up to end of May 2011.

In 2005, rabies cases were evenly spread throughout the country, even on Hiiumaa and Saaremaa islands.

### Incidence of rabies from 2006 to 2010

**Figure 1:**
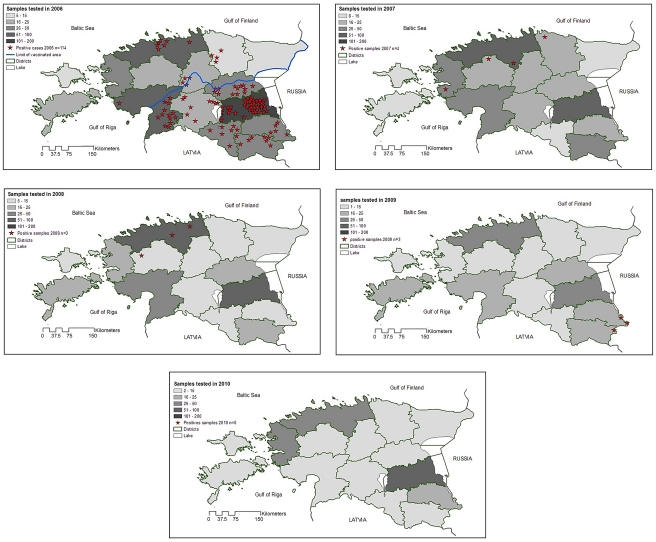
Location of rabies cases in Estonia from 2005 to 2010. Regarding the distribution of rabies cases in 2006, the area vaccinated during the autumn 2005 campaign lies above the blue line with islands.

The number of rabies cases has dropped dramatically since 2006. The decrease began following the first countrywide OV campaigns in spring 2006 (Table 2). In 2007, four rabies cases were diagnosed (two cattle, one raccoon dog and one badger), and in 2008, three rabies-positive animals (one dog, one sheep and one fox) were found during the winter-spring period. From March 2008 until the end of November 2011, only four rabies cases were reported: three rabid foxes found in May and July 2009, and one raccoon dog detected in January 2011. All four were within five kilometres of the Estonian–Russian Federation border in the South-East (Figure 1). The case recorded in January was found less than one kilometre from the Russian border, and three kilometres from one of the cases diagnosed in 2009.

Brain samples from target animals collected by hunters for OV efficacy checks (not shown in Figure 1, which presents rabies surveillance data on suspect animals and those found dead) were tested negative for the rabies virus (3,461 samples in 2008; 1,756 in 2009 and 1,750 in 2010).

### Bait uptake

From 2006 to 2010, some 2,800–3,400 samples in all were tested each year for tetracycline at the VFL.

The proportion of samples showing tetracycline line(s) ranged on average between 84% (2007, 2010) and 90% (2008), indicating that most animals had consumed the baits.

Yearly positivity rates ranged from 85% to 93% in foxes and from 82% to 88% in raccoon dogs. The overall proportion of jaws positive for the biomarker was significantly lower in raccoon dogs than in foxes: 89% in foxes and 84% in raccoon dogs (Chi2 = 67.6, *p<0.001*) (Table 3).

**Table 3:** Bait uptake and seroprotection rates in foxes and raccoon dogs from 2006 to 2010.

	Fox		Raccoon dog		Total	
Bait uptake	TC positive/total samples	% [95% CI]	TC positive/total samples	% [95% CI]	TC positive/total samples	% [95% CI]
2006	1,169/1,343	87 [84.9–88.6]	1,294/1,546	84 [81.7–85.5]	***2,463/2,889***	***85 [83.9–86.5]***
2007	1,070/1,255	85 [83.1–87.2]	1,349/1,627	83 [81.0–84.7]	***2,419/2,882***	***84 [82.5–85.2]***
2008	1,599/1,727	93 [91.6–94.1]	1,520/1,734	88 [86.0–89.2]	***3,119/3,461***	***90 [89.1–91.1]***
2009	1,033/1,131	91 [89.5–92.9]	1,617/1,880	86 [84.3–87.6]	***2,650/3,011***	***88 [86.8–89.1]***
2010	973/1,104	88 [86.0–90.0]	1,716/2,085	82 [80.6–84.0]	***2,689/3,189***	***84 [83.0–85.6]***
Total	***5,844/6,560***	***89 [88.3–89.8]***	***7,496/8,872***	***84 [83.7–85.2]***	***13,340/15,432***	***86 [85.9–87.0]]***

Sero-protection	Ab positive/total sera	% [95% CI]	Ab positive/total sera	% [95% CI]	Ab positive/total sera	% [95% CI]
2006	106/193	55 [47.6–62.0]	130/235	55 [48.7–61.7]	***236/428***	***55 [50.3–59.9]***
2007	97/286	34 [28.5–39.8]	120/317	38 [32.5–43.5]	***217/603***	***36 [32.2–40.0]***
2008	472/929	51 [47.5–54.1]	536/985	54 [51.2–58.1]	***1,008/1,914***	***53 [50.4–55.0]***
2009	310/647	48 [44.01–51.9]	513/1,091	47 [44.0–50.0]	***823/1,738***	***47 [45.0–49.7]***
2010	278/594	47 [42.7–50.9]	529/1,159	46 [42.8–48.6]	***807/1,753***	***46 [43.7–48.4-]***
Total	***1,263/2,749***	***46 [44.1–47.8]***	***1,828/3,787***	***48 [46.7–49.9]***	***3,091/6,436***	***48 [46.8–49.3]***

TC: tetracycline.

CI: Confidence Interval.

Ab positive sera: rabies antibody titer by ELISA≥0.5 EU/mL.

Tetracycline, used as a biological marker, was sought in the lower jaw of the foxes and raccoon dogs killed in vaccinated zones. The number of positive samples out of the total samples and the positivity percentage for tetracycline are reported by target species (red fox and raccoon dog).

Sera were analysed with an indirect ELISA kit (Platelia Rabies II kit, Biorad). A threshold seroconversion of 0.5 EU/mL was adopted.

**Figure 2:**
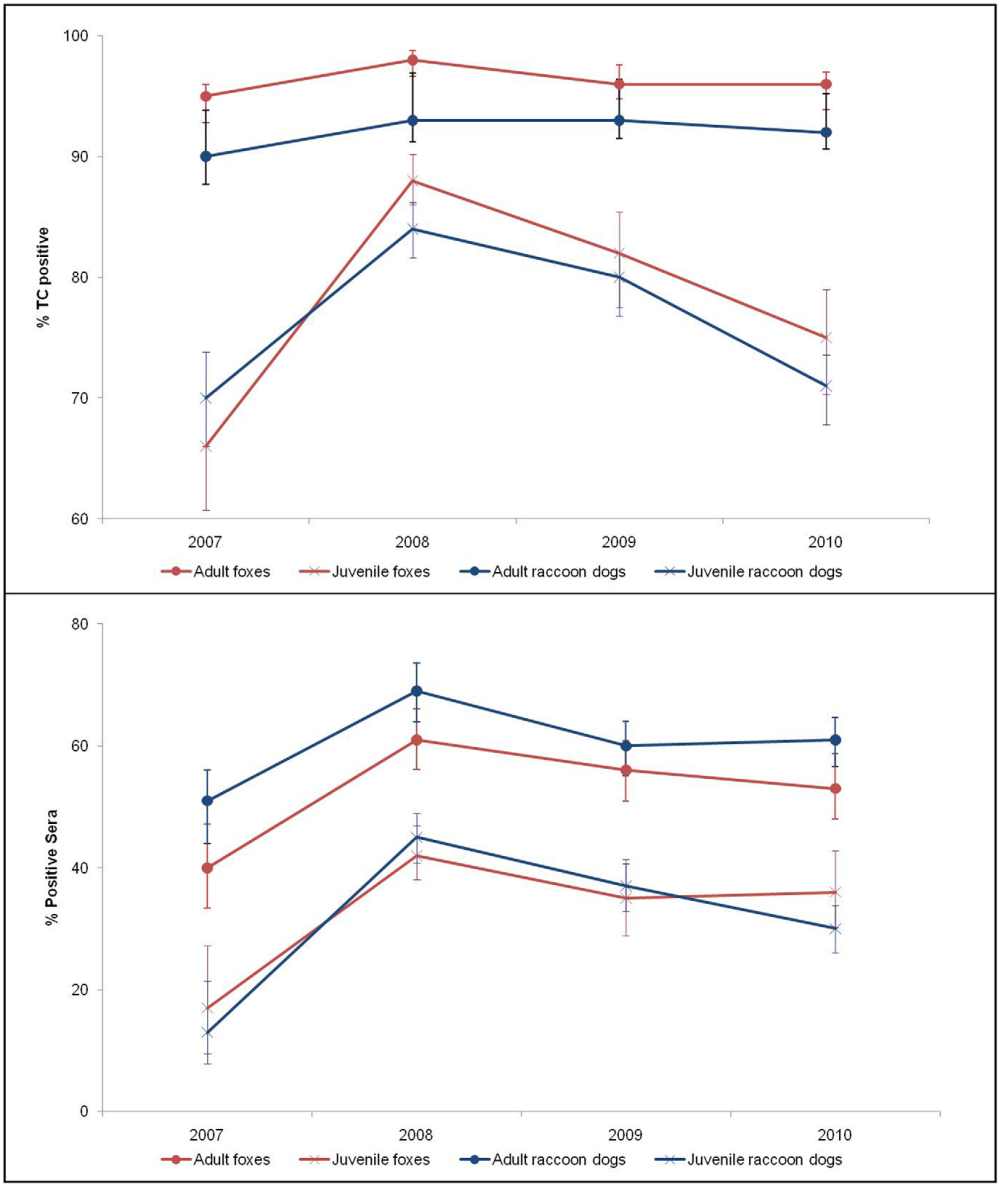
Tetracycline positivity and seroconversion in foxes and raccoon dogs (200–2010). The graphs are by age group (adult/juvenile).

Annual tetracycline positivity rates ranged from 95% to 98% in adult foxes and from 66% to 88% in fox cubs. In the raccoon dog, the tetracycline positivity rate ranged from 90% to 93% in adults and from 70% to 84% in juveniles. The overall proportion of positive jaw samples was significantly higher in adults than in juveniles for both species, with 96% in adults and 80% in juveniles in the red fox population (Chi2 = 311.2, *p<0.001*) and 92% in adults and 77% in juveniles in the raccoon dog population (Chi2 = 295.0, *p<0.01*) (Figure 2).

### Humoral response

Approximately 6,400 samples were tested for rabies antibodies at the VFL from 2006 to 2010. Annual immunisation rates ranged from 34% to 55% in foxes, and from 38% to 55% in raccoon dogs, without any significant differences between the two species (Table 3). The overall immunisation rates in the 2006–2010 period were similar in both species, with 46% in foxes and 48% in raccoon dogs (Chi2 = 3.4, non significant).

Annual fox immunisation rates ranged from 40% to 61% in adults and from 17% to 42% in fox cubs. Annual raccoon dog immunisation rates ranged from 51% to 69% in adults and from 13% to 45% in juveniles (Figure 2). Results showed overall immunisation rates significantly higher among adults than juveniles in both species, with 54% in adults compared to 37% in fox cubs (Chi2 = 63.2, *p<0.001*) and 61% in adults compared to 36% in juvenile raccoon dogs (Chi2 = 220.6, *p<0.001*).

### Phylogenetic analysis of positive samples since the initiation of OV

Of the 48 Estonian samples tested, 43 were positive by hnRT-PCR. The amplified products (589 bp) corresponding to the 43 positive RV strains belong to the lineage formed by the classical rabies virus with a bootstrap value of 85% (value corresponding to the nucleoprotein phylogenetic analysis).

**Figure 3:**
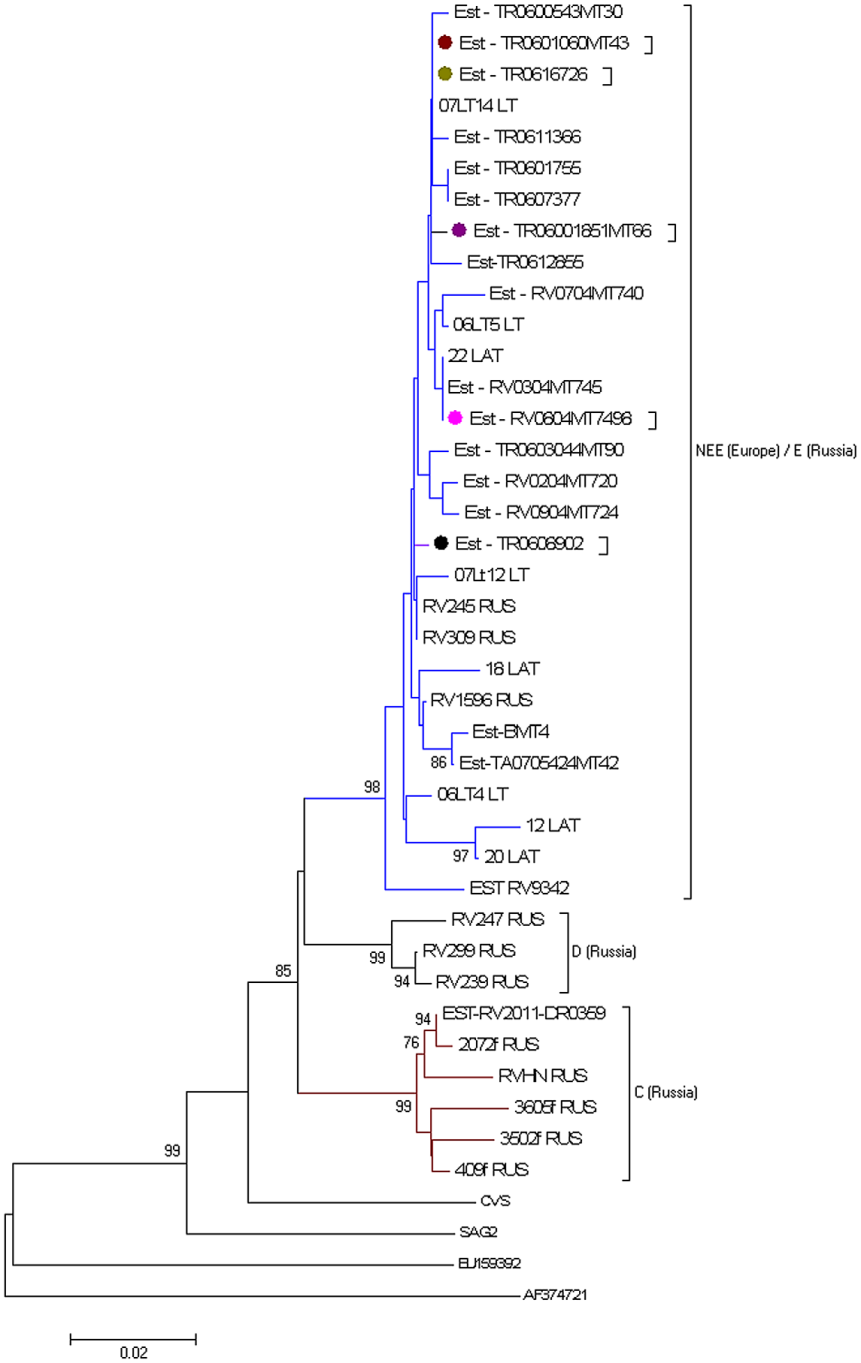
Neighbour Joining phylogenetic tree between 18 Estonian rabies virus sequences and 24 references isolates. The tree is rooted with isolates EU159392 and AF374721, used as outgroup. The phylogenetic analysis was based on the analysis of the first 400 nt of N gene using NJ method. Bootstrap values greater than 70% are shown next to the branches. Abbreviations for the phylogenetic groups (NEE, C, D and E) earlier described by Bourhy et al. [20] and Kuzmin et al. [21] are used in the text. In ◊ are shown the representative sequences of identical nucleoprotein sequences among rabies virus isolates from Estonia.

Five samples isolated in 2006 were positive by RT-PCR for the internal control, i.e. the 18S rRNA gene with an amplified product of 324 bp and negative for nucleoprotein gene amplification (589 bp). These samples were therefore investigated in the EU-RL Nancy using rabies reference diagnostic methods. All five samples were found negative by FAT, cell culture and mouse inoculation tests. 17 out of 18 Estonian sequences could be placed in one lineage (bootstrap of 98) belonging to the North East Europe (NEE) group of rabies virus [20]. The latter is mainly composed of the 17 isolate sequences from Estonia, in addition to reference sequences from Latvia (n = 4), Lithuania (n = 4) and from Russia (n = 3), representative of the group E (North-western part of Russia), earlier described by Kuzmin et al. [21] (Figure 3). The group NEE was also linked with 3 sequences of group D, representative of the centre of the European part of Russia [21]. The 17 Estonian sequences exhibited 99% identity among them and 98.4% identity against the 9 published reference European sequences (Estonia, Latvia and Lithuania) belonging to the group NEE. A 98.7% identity was also shown between the 17 Estonian sequences and the 3 Russian sequences forming the group E (RV309, RV245 and RV1596).

The phylogenetic tree also showed that the viral strain (Est-RV2011-DR0359) isolated in 2011 in Estonia is placed in the second group C, representative of Russia already described [21]. This group, constituted by 5 referenced Russian isolates and isolate Est-RV2011-DR0359, formed a solid cluster with a significant bootstrap support value (99%). 98.4% identity was also shown between the 2011's sample and the five Russian isolates (Figure 3).

The analysis of 43 Estonian sequences showed that all the amplified isolates belong to the same clade of classical rabies virus, which clearly differs from that of the rabies virus strain used for orally vaccinating wildlife (SAG2).

A sequence analysis of N (400 nucleotides, positions 71 to 747) and G genes (690 nucleotides, positions 3911 to 4600) comparing isolates TR0608902 (raccoon dog isolated in 2006) and TR0603634 (red fox isolated in 2006) using the Vector NTI and BioEdit software showed a perfect nucleotide similarity with 100% identity (data not shown). The same 100% nucleotide identity was also revealed when the G gene was compared with two other samples isolated in 2006: red fox (TR06-12855) and raccoon dog (TR06-11366) (data not shown).

### Costs

#### Costs of oral vaccination of wildlife in Estonia (2005–2010)

In 2003, an OV programme was initially planned for a three-year period in the framework of a PHARE project. The programme was reduced to two years and accepted in the framework of the Transition Facility project “Minimalisation of the number of rabies cases among wild and domestic animals in Estonia”. The project was finally kicked off in autumn 2005, but was limited to one vaccination campaign only. Since 2006, the rabies elimination programme has been co-financed by the EU and the Estonian state budget.

The costs of OV in Estonia from 2005 to 2010 are detailed in Table 4. As expected, vaccine supply represented the main cost (62%), followed by the cost of aerial distribution (19%) and costs for parenteral vaccination of domestic animals (8%). The total number of vaccinations in domestic animals has continuously decreased since 2005 (data not shown), the increase in costs in 2008 corresponding to a 25% increase in the vaccination procedure. Post-exposure prophylaxis costs represented 3% of total costs (Table 4). The number of post-exposure courses of treatment has more than halved since 2005 (1154 courses of treatment in 2005 versus 556 in 2009), with vaccine and immunoglobulin costs increasing 10–12% each year (with a 16% increase in 2008).

**Table 4:** Main costs of the rabies control programme in Estonia from 2005–2010.

	Main costs of OV of wildlife in Estonia in 2005–2010 (in Euros taxes included)						
	2005	2006	2007	2008	2009	2010	*Total*
Vaccine supply	422,453.4	1,453,213.9	1,350,385.8	1,362,032.5	1,396,917.1	1,396,917.1	**7,381,919.8**
Aerial distribution	145,010.0	409,034.0	409,034.0	428,208.0	428,208.0	462,464.7	**2,281,958.7**
Samples collection	15,655.8	23,524.6	39,875.4	48,911.4	45,519.5	44,544.2	**218,030.9**
Post-vac. laboratory tests	19,614.5	27,055.5	45,771.8	197,559.0	152,278.7	154,406.6	**596,686.1**
Awareness campaign	24,999.0	28,866.1	33,245.7	9,830.1	12,524.7	10,833.1	**120,298.7**
Other costs	18,377.2	3,797.5	23,641.2	20,738.8	19,877.8	20,441.8	**106,874.3**
Parenteral vaccinations	170,736.3	163,140.7	156,305.4	181,063.8	171,399.0	108,038.0	**950,683.2**
Post-exp. prophylaxis	61,355.0	49,212.0	47,934.0	60,716.0	52,408.0	57,116.0	**328,741.0**
***Total***	***878,201.2***	***2,157,844.3***	***2,106,193.3***	***2,309,059.6***	***2,279,132.8***	***2,254,761.5***	***11,985,192.7***

#### Comparison of rabies incidence and funding allocated by the European Commission for rabies control programmes in Estonia, Latvia and Lithuania

In Latvia, the first attempts at OV over areas >4,000 km^2^ were programmed from 1992 to 2000 (443,710 baits distributed) but neither regularly nor accurately (manual distribution) [22]. From 2001 to 2003, vaccination was extended to the whole Latvian territory [23], still with manually-distributed vaccine baits. Since 2005, OV campaigns have been carried out using fixed-wing aircraft or helicopters at a density of 23–25 baits/km^2^ in spring and in autumn over the whole country except in 2005, when the vaccination area was reduced to half of the territory and in 2008, when the vaccinated area was reduced to 8,800 km^2^ in the spring and 40,511 km^2^ in the autumn [23]. The SAD Bern and SAD B19 vaccine strains were used. Since 2009, only the SAD B19 vaccine strain has been used.

In Lithuania, a five-year OV programme was conducted from 1996 to 2000 [24] by manual or aerial distribution twice a year, over areas ranging from 4,000 to 15,000 km^2^. Three vaccine strains were used: SAG1, SAD Bern and SAD B19. A total of 900,000 baits were distributed [24]. Oral vaccination was discontinued from 2001 to 2006 in Lithuania. From 2006 to late 2010, a new vaccination strategy was adopted. Baits were distributed at a density of 20 baits/km^2^ throughout the territory in spring and autumn from aircraft [25]. SAD Bern baits were used in 2009 and 2010.

**Figure 4:**
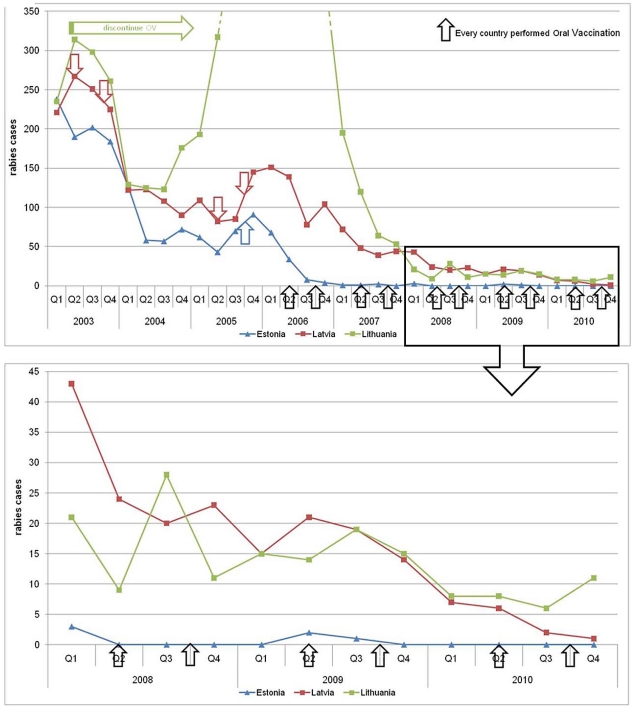
Evolution of rabies cases from 2003–2010 in Latvia, Lithuania and Estonia. A special focus is on the 2008–2010 period. The arrows correspond to the different vaccination campaigns.

**Figure 5:**
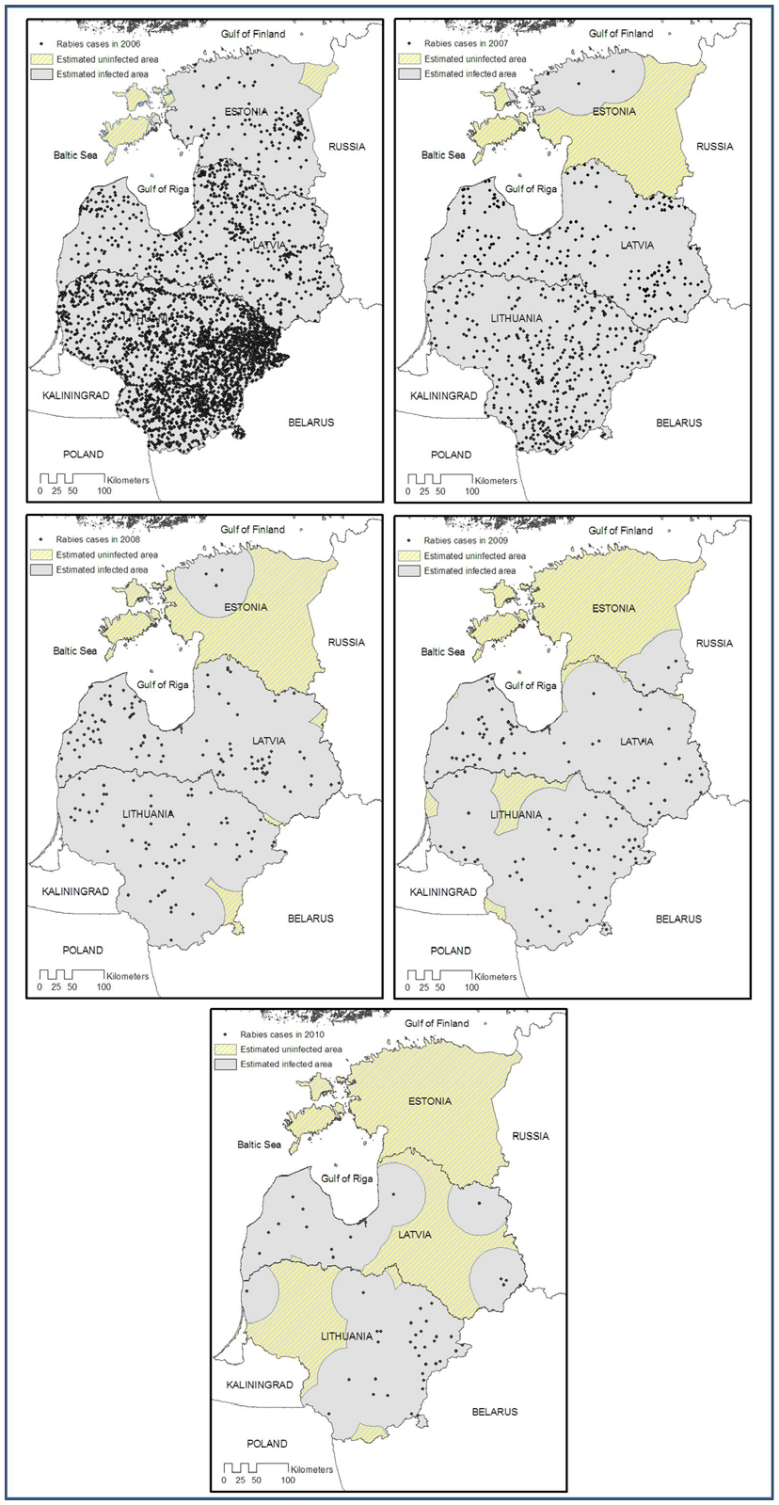
Location of rabies cases from 2006 to 2010 in Estonia, Latvia and Lithuania. For each map, representation of areas with and without rabies cases (“buffer” zone of 50 km around each positive case).

The evolution of rabies incidence from 2003 to 2010 in Estonia, Latvia and Lithuania is presented in Figures 4 and 5. Arrows correspond to the different vaccination campaigns.

Since 2006, the number of rabies cases has rapidly decreased in all three Baltic States. In 2010, 33 and 16 rabies cases were detected in Lithuania and Latvia respectively, spread throughout their territory (Figure 5).

The focus on the 2008–2010 period showed that after an initial drop in the three Baltic States, a few rabies cases have still been identified in Latvia and Lithuania, while rabies has been nearly eradicated in Estonia since summer 2009 (Figure 4).

Table 5 presents for each Baltic State the number of baits distributed, funding allocated by the EC from 2006–2010 and the vaccine strain used (if published) for the 2006–2011 period. The financial contribution of the EC covers about 50% of the estimated cost of purchasing and distributing vaccine baits in addition to the cost of laboratory testing. From 2011, EC funding cover 75% of the estimated cost of rabies control programmes.

**Table 5:** Overview of the cost of OV in Estonia, Latvia and Lithuania from 2006 to 2011.

Country/Year (y)	Vaccine strain	Rabies cases	Baits nb distributed (A)	Vaccinated area (B) in km^2^	A/B in baits/km^2^	EC funding (C) in Euros	Uninfected area in km^2^	Newly uninfected area^a^ (D) in km^2^	C(y−1)/D(y) in Euros/km^2^
**ESTONIA**									
2006	SAG2	114	1,809,080	45,000	40,2	990,000	5,825	/	/
2007	SAG2	4	1,716,800	45,000	38,2	925,000	32,202	26,377	37,5
2008	SAG2	3	1,720,400	45,000	38,2	860,000	35,578	3,376	274,0
2009	SAG2	3	1,720,000	45,000	38,2	870,000	38,078	2,500	344,0
2010^c^	SAG2	0	1,720,000	45,000	38,2	1,360,000	44,949	6,871	126,6
*2011* c			*376,000*	*9,400*	*40,0*				
**Period 2006–2010**		**124**	**8,686,280**		**38.6 (M)**	**5,005,000**		**39,124**	**93.2** d
***Period 2006–2011***			***9,062,280***		***38.8 (M)***				
**LATVIA**									
2006	SAD B19 and SAD Bern	472	3,372,000	64,000	52,7	650,000	0	/	/
2007	SAD Bern	203	3,351,600	64,000	52,4	1,200,000	0	0	/
2008^b^	SAD B19 and SAD Bern	110	919,200	49,326	18,6	700,000	728	728	1648,4
2009	SAD B19	69	2,980,800	64,000	46,6	850,000	1,128	400	1750,0
2010^c^	SAD B19	16	3,200,000	64,000	50,0	1,400,000	23,761	22,633	37,6
*2011* c			*3,200,000*	*64,000*	*50,0*				
**Period 2006–2010**		**870**	**13,823,600**		**44.1 (M)**	**4,800,000**		**23,761**	**143.1** d
***Period 2006–2011***			***17,023,600***		***45.0 (M)***				
**LITHUANIA**									
2006	?	2232	2,100,000	65,000	32,3	600,000	0	/	/
2007	?	432	2,600,000	65,000	40,0	600,000	0	0	/
2008	?	69	2,600,000	65,000	40,0	700,000	1,919	1,919	312,7
2009	SAD Bern	63	2,600,000	65,000	40,0	500,000	6,164	4,245	164,9
2010^c^	SAD Bern	33	2,600,000	65,000	40,0	540,000	19,686	13,522	37,0
*2011* c			*2,600,000*	*65,000*	*40,0*				
**Period 2006–2010**		**2829**	**12,500,000**		**38.5 (M)**	**2,940,000**		**19,686**	**121.9** d
***Period 2006–2011***			***15,100,000***		***38.7 (M)***				

a : between (y) and (y−1).

b : 1 OV campaign.

c : 75% of EC funding.

d : It is assumed that the effect of OV is observable at year y+1. This ratio corresponds to the EC funding from 2006 to 2009/newly uninfected area from 2007 to 2010.

(M): Mean value.

This table shows the number of baits used, the funding allocated for rabies prevention by the European Commission (EC) “approving annual and multi-annual programmes and the financial contribution”, the area vaccinated, the uninfected and newly uninfected areas and the type of vaccine used. Different ratios were calculated : Number of baits distributed from 2006–2011 per country per vaccinated area in km^2^. EC funding over the 2006–2010 period/newly uninfected areas in the country in km^2^.

From 2006 to 2011, two campaigns of vaccination were performed each year in the three countries except for Latvia in 2008, resulting in a total number of baits distributed of approximately 9.1 million in Estonia, 17.0 million in Latvia and 15.1 million in Lithuania. The ratio of baits to vaccinated area is the lowest in Estonia and in Lithuania (39 baits/km^2^), then in Latvia (45 baits/km^2^) over the 2006–2011 period. Figure 5 presents for each Baltic State the rabies situation from 2006–2010 along with areas newly uninfected. It should be noted that the data for rabies cases were taken from the Rabies Bulletin Europe and positive cases reported on a regional scale [13], whereas the data in figure 1 are reported on a municipality scale. In Estonia, areas in the North East were reported to have been freed of rabies from 2006. Rabies-free areas increased dramatically every year and remained rabies-free up to 2010, when the whole territory was considered rabies-free. In Latvia and Lithuania, the whole territory was still infected until 2010, when approximately 30% of the two countries had newly uninfected areas.

From 2006 to 2010, cumulated EC funding reached 5.0×10^6^ euros for Estonia, 4.8×10^6^ euros for Latvia and 2.9×10^6^ euros for Lithuania (Table 5). The ratio EC funding/area freed from rabies is the lowest for Estonia (93 euros/km^2^), followed by Lithuania (122 euros/km^2^) and Latvia (143 Euros/km^2^).

## Discussion

In autumn 2005, Estonia initiated oral vaccination programmes to control rabies. The organisation and implementation of these rabies vaccination campaigns comply with the recommendations of the European Commission [10].

The efficacy of the OV campaigns in Estonia was assessed by monitoring rabies prevalence in mammals, bait consumption and the immunisation rates in fox and raccoon dog populations. Rabies prevalence was the primary efficacy criterion. Passive surveillance was extended throughout the country by analysing field samples from domestic and wild animals suspected of rabies in addition to mammals found dead [13].

Since the beginning of OV programmes, the incidence of rabies in Estonia has dropped dramatically, with only three to four cases reported annually from 2007 to 2009. Since March 2008, no rabies cases have been detected in Estonia with the exception of three cases reported in summer 2009 and one case in January 2011 in areas close to the South-Eastern border. This last case was identified as a spill-over from Russia and measures have already been adopted to increase rabies awareness in this county. It must be noted that this drop in rabies incidence was observed despite the recent expansion of fox and raccoon dog populations in Estonia (data not shown).

Oral vaccination campaigns were monitored from late July of the OV year to the following March by collecting head and serum samples from foxes and raccoon dogs. Autumn campaigns target both adults and juveniles, while spring campaigns target mainly adults, because fox cubs are usually born from 15 March to 15 April, and raccoon dogs in May [26], [27]. As we expected the animal's age to have an effect on bait uptake and immunisation levels as previously demonstrated [28], we determined the age of all the animals collected from 2007 on.

The proportion of tetracycline-positive samples was high and stable in adults of both target species (≥95% in foxes and ≥90% in raccoon dogs). These results confirmed the efficacy of the aerial distribution strategy and the attractiveness of SAG2 baits for both raccoon dogs and foxes [29], [30]. In juveniles, bait uptake was significantly lower than in adults, ranging from 66% to 88% in fox cubs, and from 70% to 84% in young raccoon dogs. Few studies have evaluated the effectiveness of OV according to the age of the target species [28], [31]. Bruyère et al [28] reported the difficulties in reaching juveniles during baiting campaigns, especially when spring campaigns were conducted in April instead of late May. The tetracycline positivity rates obtained in Estonia are consistent with those reported in foxes during the 1994–2001 period in France with values of 86% after the spring and autumn campaigns in adults versus 63% and 79% after the spring and autumn campaigns in fox cubs [32]. An investigation of diurnal and nocturnal movement patterns of juvenile foxes in the UK suggested that this low bait uptake is explained by their reduced ranging behaviour and the concentration of their activity at secure sites (“rendezvous sites”) [33]. Unless baits are distributed at these secure sites, the probability of vaccinating cubs before the dispersal period is therefore limited. New strategies should be tested to improve the efficiency of OV in cubs, which constitute over half of the target population.

Immunisation rates were similar in both species, ranging in adults from 40% to 61% for foxes and from 51% to 69% for raccoon dogs. In 2007, immunisation rates were significantly lower than those observed in 2006 and from 2008 to 2010. This result may be explained by the earlier dates chosen for the spring 2007 campaign (22 April to 15 May) compared to vaccination periods since 2008 (15 May to early June). This had a clear impact on the immunisation rates observed in young animals after the spring campaign. The rate of immunisation was 45% in adults versus 8% in young animals for samples collected from July to November 2007 after the spring campaign earlier that year. In contrast, 47% of adults versus 43% of young animals had sero-converted after the autumn campaign of 2007 (samples collected from December 2007 to March 2008) (data not shown). From 2008, immunisation rates in adults from both species were close to 60%, except in foxes in 2010. This level corresponds to the vaccination coverage (60%–70%) estimated to be sufficient to break the rabies cycle [34]. Immunisation rates were significantly lower in juveniles, with values ranging from 17% to 42% in fox cubs, and from 13% to 45% in young raccoon dogs. Similar data have also been obtained in France, with lower immunisation rates in fox cubs than in adult foxes [28]. Immunisation rates were significantly lower than tetracycline positivity rates in both adults and young animals, the difference being more marked in juveniles. Several hypotheses may explain these discrepancies. First, the bait casing may be ingested while the capsule containing the vaccine is not. Brochier et al. [35] postulated that cubs may chew the baits without puncturing the vaccine capsule. When they are not hungry, foxes hide their baits to eat later, so the vaccine may be inactivated [36]. Fluorescence in teeth may be seen without any tetracycline absorption or animals may find sources of tetracycline other than vaccine baits: in France, 9.5% of foxes (34/357) were tetracycline-positive in non vaccinated areas (unpublished data). Other hypotheses have been already given [28]. Vixens feed their weaning cubs by regurgitation, and while regurgitated baits still contain tetracycline, the SAG2 strain is likely to be destroyed by gastric acidity. Contact between the vaccine suspension and the oro-pharyngeal mucosa may sometimes be insufficient for immunisation. The production of antibodies may be transient or absent, or reach a low titre. In this last case, the sensitivity of the test used is a critical factor. Recently, Knoop et al. [37] showed that the Bio-Rad immunoassay used for the titration of rabies antibodies has a poor sensitivity using fox field samples from OV areas (32.4% as compared to the Rapid Fluorescent Focus Inhibition Test [RFFIT]). This study showed that antibody titres expressed in EU/mL were 2 to 5 times lower than those obtained with sero-neutralisation assays resulting in a constant underestimation of titres with this immunoassay. These results are consistent with those of a previous investigation conducted at the EU-RL in Nancy [38]. Consequently, multiple-vaccinated adults with high antibody titres may thus be more easily found positive by an ELISA test than primo-vaccinated juveniles.

International organisations also recommend that “all rabies virus isolated should be typed in areas where attenuated rabies virus vaccines are used, in order to distinguish between vaccine and field virus strains” [10]. Phylogenetic analysis demonstrated that all 43 field isolates from Estonia found positive by hnRT-PCR belong to the classical rabies virus (genotype 1) and are all closely related. This shows that no vaccine-induced rabies cases occurred and all positive animals were infected with wild rabies strains present in Estonia. Of the 48 samples detected positive by FAT in Estonia, five samples were shown to be negative by reference techniques and hnRT-PCR at the EU-RL in Nancy. This discrepancy may be due to a degradation of the RNA (storage conditions, transportation to France…).

Our phylogenetic study showed that rabies strains isolated in the 2004–2010 period from the two main wildlife reservoirs in Estonia—the raccoon dog and red fox populations—belong to the same group, NEE. The same phylogenetic results were observed in Lithuania by comparing various isolates (raccoon dogs and red foxes) against published isolates from neighbouring countries [39]. In our study, the comparison of nucleoprotein and glycoprotein gene sequences between two foxes and two raccoon dog samples isolated in 2006 showed 100% identity. The same perfect identity was also shown between one isolate (raccoon dog) from 2006 and three strains (3 red foxes) isolated in 2009 in Estonia. This genetically close relationship between the isolates strongly suggests that the variant circulating in fox and raccoon dog populations have the same origin. As suggested by Bourhy et al. [40], dogs may have served as an early vector for interspecies rabies virus transmission, generating viral lineages that then spread to other taxa. Phylogenetic data suggest the hypothesis of single rabies epidemics in red foxes and raccoon dogs in Northern Europe as previously interpreted [39]. Our phylogenetic study showed that the latest positive case that occurred in 2011 in Estonia belongs to the group C of Russia, suggesting a spill-over from Russia.

The three Baltic States—Estonia, Latvia and Lithuania—share similar features. Rabies incidence had increased at the beginning of the 2000s, in particular in wildlife. The main vectors and virus reservoirs are the red fox and raccoon dog populations [22], [41]. Phylogenetic analyses have shown that rabies viruses from Estonia, Latvia and Lithuania belong to the same clade [22], so it was considered important to compare the evolution of rabies in the three Baltic States.

Since vaccination campaigns were incomplete or absent before 2006 for all three countries, we compared the number of baits distributed and the EC's financial contribution in the three Baltic States since 2006.

Rabies incidence has rapidly decreased since 2006 in the three Baltic States following the implementation of oral vaccination programmes in those countries from 2006. However, while no rabies cases have been recorded in Estonia since summer 2009 (except one case in January 2011 within one kilometre of the Eastern border with Russia), rabies cases were still diagnosed in Latvia and Lithuania in 2010. In France, similar results were obtained when comparing the efficacy of SAD B19 and SAG vaccines in the field: results demonstrated a faster, more durable decrease in rabies incidence when using SAG baits [42]. Despite OV campaigns since 2006 in all infected areas, no area has been successfully freed of rabies in Latvia and Lithuania. It should be noted that rabies incidence differed in the three countries during the period considered for comparison. The period following 2006 was chosen as it corresponded to the start of large scale oral vaccination in all three countries. Lithuania in particular recorded a huge number of cases in 2005 and 2006 then a dramatic drop as early as 2007. Other experiences in Western European countries [4] have shown that the time required to eliminate rabies using oral vaccination campaigns is not correlated with the number of cases in infected areas.

Indeed, the effectiveness of vaccination campaigns relies on three critical elements, i.e. the vaccination strategy, the vaccine and bait used, and the geographical situation of the countries. A tender procedure was used in all three countries for the procurement and aerial distribution of vaccine baits.

All three Baltic States used modified-live vaccines derived from the original SAD (Street Alabama Dufferin) strain isolated in a naturally rabid dog in the U.S.A. The SAD Bern strain is a cell-culture-adapted derivative of SAD. Different vaccines have also been derived from SAD Bern: SAD B19 after attenuation on cloned BHK21 cells, SAG1 and SAG2 vaccines after one or two successive mutations of arginine 333, associated with reduced rabies virus pathogenicity and selected by using monoclonal antibodies [9]. SAG2 was used in Estonia from the very first campaign in autumn 2005 in the Northern part of the country. Both SAD Bern and SAD B19 vaccines have been used in Latvia, SAD B19 being used since 2009. In Lithuania, SAD Bern has been used since 2009 (no information from 2006 to 2008). SAG2 baits have been shown to be stable in the environment, resistant to mechanical forces, water and heat (http://www.ema.europa.eu/docs/en_GB/document_library/EPAR_-_Scientific_Discussion/veterinary/000043/WC500067900.pdf). In contrast, SAD Bern baits have been reported to lack stability in field conditions, with deformation and disintegration of bait casing and loss of vaccine titre depending upon weather conditions such as sunlight and rain [25].

Since 2006, the vaccination campaigns using aerial distribution have been conducted in spring and autumn in the three Baltic States. The bait density per campaign in Estonia and Lithuania was 20 baits/km^2^, and 23–25 baits/km^2^ in Latvia [23], [25]. A spatial simulation showed that a higher bait density (over 20 baits/km^2^) did not reduce the number of under-baited fox groups, had no beneficial effect on the success of OV and wasted resources [43]. The distance between flight paths for the aerial distribution was 600 m in Estonia, as recommended [10], and 1000 m in both Latvia and Lithuania [23], [44]. Although this smaller mesh slightly increased the cost of the flights, the flight line spacing selected in Estonia may increase bait access by the target species. Since autumn 2010, the distance between flight paths was reduced to 500 m in Latvia, and the number of vaccine baits distributed was increased to 26.5 baits/km^2^ in areas of the country where vaccination was interrupted because of tendering problems [45]. Tetracycline positivity rates are higher in Estonia than in Lithuania or Latvia in both target species. This difference in bait uptake is more marked in raccoon dogs than in foxes. Tetracycline positivity rates in foxes were 93% and 91% in Estonia in 2008 and 2009 respectively, 79% in Lithuania in 2009 [44] and 74% and 75% in Latvia in 2008 and 2009 respectively [23], [46]. Tetracycline positivity rates in raccoon dogs were 88% and 86% in Estonia in 2008 and 2009 respectively, 58.3% in Lithuania in 2009 [44] and 50% and 65% in Latvia in 2008 and 2009 respectively [23], [46]. Bait uptake levels in Estonian fox and raccoon dog populations in 2009 were statistically higher than those obtained in Latvia (no statistical analysis was carried out for Lithuania because the number of animals tested was not available). Apart from the baiting distribution strategy, a lack of bait palatability to raccoon dogs may be critical. Surprisingly, to our knowledge very few data are available regarding both the attractiveness and efficacy of available oral vaccines for the raccoon dog model under experimental conditions. Furthermore, there are no published data on the minimal titres of SAD Bern vaccine required to properly immunise foxes and raccoon dogs. Prior to their use in Estonia, the safety, attractiveness and efficacy of SAG2 baits were demonstrated in caged raccoon dogs according to European guidelines [8]. All animals had seroprotective neutralising antibody titres after ingesting the baits, and all animals vaccinated in this way proved to be protected after a virulent challenge performed six months after OV, whereas all the control animals succumbed to rabies [30]. Since raccoon dogs are one of the two major wildlife vectors and a reservoir in Northern European wildlife, raccoon dogs are a priority target for rabies control strategies in the Baltic States. Efficacy and safety data should be compulsory for all vaccine baits claiming to be suitable for use in that species.

The geographical situation is also an important factor for rabies control, as wild animals ignore borders. The three Baltic States are bordered by rabies-contaminated countries: Estonia with Russia and Latvia; Latvia with Russia, Belarus and Lithuania; and Lithuania with Latvia, Belarus, Poland and Russia (Kaliningrad Oblast). In Poland and the Kaliningrad region, wildlife OV programmes have been conducted throughout the territory since 2002 [47] and 2007 respectively [45]. The disease is endemic in Belarus and Russia, and the EU will provide funding, under certain conditions to be fulfilled by the countries concerned, for cross-border vaccination of Lithuania and Estonia. The land borders for Latvia (1,150 km) and Lithuania (1,273 km) are approximately twice those of Estonia (633 km). The West and North of Estonia is bordered by the sea, while the coastline in Latvia is shorter and in Lithuania even shorter. It should be noted that the South of Estonia has been rabies-free since 2007, demonstrating the importance of cooperation between neighbouring countries during vaccination campaigns. Despite a favourable situation in South Estonia for the past four years in all large areas bordering its 339–km-long Southern border, Latvia still reported cases close to this border with Estonia (one case recorded in 2010 was located approximately 10 km from the border). Natural barriers are obviously important to prevent the reintroduction of rabies from neighbouring countries. However, this factor may explain the presence of isolated cases close to the land borders, as observed in summer 2009 and in winter 2011 in Estonia, but not the persistence of scattered cases observed on Latvian and Lithuanian territory.

Few analyses have been published regarding the cost-effectiveness of OV among wildlife for assessing rabies elimination. A study has estimated the costs associated with rabies epidemics (vaccination of domestic animals, reinforcement of epidemiological networks, support for rabies diagnosis, animal and economic losses, clinical observation of animals which have bitten humans, prophylactic vaccination and post-exposure treatment of humans) with those of oral vaccination campaigns (cost of vaccine baits and their delivery, follow-up to ensure the efficacy of vaccination) [48]. The benefits in terms of cost of wildlife vaccination were obtained after the fourth year of the programme, the highest costs being the preventive vaccination of pets and prevention in humans. In Estonia, an analysis of costs revealed that 62% of costs are channelled into purchasing oral vaccines, whereas costs associated with the vaccination of domestic animals and post-exposure prophylaxis represented 12% of total costs. In 2010, a 35% decrease in costs for parenteral vaccines for domestic animals was reported due to the successful results of rabies control in wildlife. It should be noted that the decrease in vaccination number concerned livestock in particular; furthermore, a new regulation dated July 2009 authorised a booster vaccination every two years instead of annually. The number of post-exposure courses of treatment has also decreased since 2005. We hypothesise for the coming years a continued decrease in costs for most of the expenses required for rabies control (vaccine and distribution material and services, parenteral vaccination of domestic animals, post-exposure prophylaxis and laboratory analysis).

It was also considered worthwhile to compare the cost-effectiveness of OV in the three Baltic States. We selected the 2006–2011 period because before then, no OV campaigns had been regularly conducted throughout these countries. Data were obtained using the same source of published reports (EC funding programmes). Our analyses showed that the mean yearly number of baits used per square kilometre of vaccinated area was the lowest in Estonia and in Lithuania (39 baits/km^2^), followed by Latvia (45 baits/km^2^). A higher bait density was used in Latvia. Furthermore, during the 2006–2010 period, a comparison between EC funding and the areas newly uninfected, i.e. the cost required to free an area from rabies, was also the lowest for Estonia (93 euros/km^2^), followed by Lithuania (122 euros/km^2^) then Latvia (143 euros/km^2^). It should be noted that the cost of the SAG2 baits used in Estonia is higher (around 0.83 euros per bait in 2010 for SAG2) than that of SAD Bern/SAD B19 (0.50 euros per SAD Bern/SAD B19 bait based on EU price indications in different programmes). Rabies cases were still scattered throughout Lithuania and Latvia in 2010, whereas rabies incidence dropped quickly and dramatically as early as 2006 in Estonia with fewer baits and without any re-infection of freed areas (except the small part at Eastern border). The Estonian strategy, leading to rabies elimination, is thus clearly more advantageous in terms of cost-effectiveness.

It is costly to keep a country rabies-free when neighbouring countries are still infected. Freuling et al. [49] have evaluated the cost of a vaccination belt to prevent the re-infection of EU countries by infected non-EU countries. Based on two campaigns per year, a bait density of 30 baits/km^2^ per campaign and a distribution by fixed-wing aircraft with a flight path distance of 500 m, the annual cost would come to 10–16 M euros [50]. Despite the expansion of the red fox and raccoon dog populations [1], Estonia's experience clearly demonstrated that a density of 20 baits/km^2^ is sufficient to control rabies.

**Figure 6:**
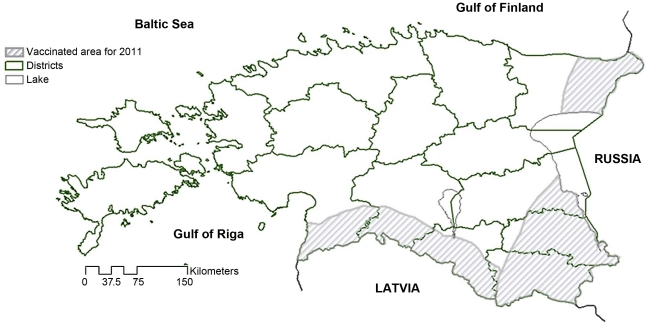
Location of oral vaccination “buffer” zones. Are detailed the “buffer zones between Estonia and Russia (1,521 km^2^ in the North-East and 4,318 km^2^ in the South) and between Estonia and Latvia (3,486 km^2^) in 2011.

It is generally recommended to perform four vaccination campaigns (i.e. two years of OV) after the last rabies case diagnosis [10], [50]. In Estonia, a buffer zone was established in 2011 (covering a total of 9,325 km^2^) with Russia and Latvia (Figure 6). The width of the buffer zone between Estonia and Russia was 30 km in areas where natural barriers exist, such as Lake Peipsi and the Narva river, or 50 km in the event of a mainland border. The width of the buffer zone between Latvia and Estonia was 20 km in most cases, but extended to 40 km in areas where rabies cases were diagnosed near the borders. The EU co-financing in Estonia for 2012 will include routine vaccination in buffer-zone (the same than that of 2011) twice a year and also emergency funds for vaccination in the event of the occurrence of residual rabies foci (emergency vaccination in 8,000 km^2^). OV will be pursued twice a year in these higher-risk areas to maintain a sufficient level of immunity among raccoon dogs and foxes. A bait density of 20 baits/km^2^ should be sufficient. Previous examples of successful long-term elimination of rabies in Western Europe have demonstrated that rabies control also requires coordination with neighbouring countries [10]. Effective collaboration with Latvia and Lithuania, based on annual meetings on rabies issues and day to day contacts for coordination of vaccination activities, is an important element of the program's success and will be maintained. A partnership with Russia to create an EU-financed vaccination belt between the two countries is being considered. Continuous passive rabies surveillance will be carried out throughout the Estonian territory and will be reinforced along the borders with Russia and Latvia. In well identified high risks areas, active surveillance could be used as a complement to passive surveillance [51]. OV efficiency will be verified only in the buffer zone. Should there be a re-emergence of rabies cases, OV will be rapidly initiated around the site of the event.

### Conclusion

Estonia has implemented a rabies control program since autumn 2005. Rabies cases have not been detected for almost 4 years, with the exception of four cases all very close to the South-Eastern border. The example of rabies control in Estonia illustrates how rabies may be quickly and successfully eliminated through successive oral vaccination campaigns among wildlife by strictly following current recommendations from the EC [10], WHO [6] and the OIE [50]. The vaccination strategy and a potent vaccine are key factors to success.

For Estonia to reach a rabies-free status, oral vaccination campaigns among wildlife in buffer zones close to infected neighbouring countries were carried out in 2011 and will be continued until these infected countries also become rabies-free.

## References

[1] Niin E, Laine M, Guiot AL, Demerson JM, Cliquet F (2008). Rabies in Estonia: situation before and after the first campaigns of oral vaccination of wildlife with SAG2 vaccine bait.. Vaccine.

[2] Anonyme (2000). Communicable disease statistics in Estonia. Part 7: 2000.. Health Protection Inspectorate, Tallinn.

[3] Bugnon P, Breitenmoser U, Peterhans E, Zanoni R (2004). Efficacy of oral vaccination in the final stage of fox rabies elimination in Switzerland.. J Vet Med B Infect Dis Vet Public Health.

[4] Aubert MFA, Cliquet F, Smak JA, Brochier B, Schon J, King A, Fooks AR, Aubert M, Wandeler AI (2004). Rabies in France, the Netherlands, Belgium, Luxembourg and Switzerland. Historical perspective of rabies in Europe and the Mediterranean Basin.

[5] Capello K, Mulatti P, Comin A, Gagliazzao L, Guberti V (2010). Impact of emergency oral rabies vaccination of foxes in northeastern Italy, 28 December 2009–20 January 2010: preliminary evaluation. Eurosurveillance.

[6] Anonyme (2005). WHO expert consultation on rabies.

[7] Niin E, Barrat J, Kristian M, Demerson JM, Cliquet F, Dodet B SA, Pastoret PP, Lombard M (2006). First oral vaccination of wildlife against rabies in Estonia.. First international conference on rabies in Europe. 2006/08/02 ed.

[8] Anonyme (2008). Rabigen SAG2, live attenuated rabies virus, SAG strain.. European Medicines Agency.

[9] Lafay F, Benejean J, Tuffereau C, Flamand A, Coulon P (1994). Vaccination against rabies: construction and characterization of SAG2, a double avirulent derivative of SADBern.. Vaccine.

[10] Anonyme (2002).

[11] Anonyme (2007).

[12] Dean DJ, Abelseth MK, Atanasiu P, Meslin FX, Kaplan MM, Koprowski H (1996). The fluorescent antibody test.. Laboratory techniques in rabies. 4th ed. ed.

[13] Cliquet F, Freuling C, Smreczak M, van der Poel WHM, Horton D (2010). Development of harmonised schemes for monitoring and reporting of rabies in animals in the European Union..

[14] Johnston DH, Joachim DG, Bachmann P, Kardong KV, Stewart REA, Nowak M BJ, Obbard ME, Malloch B (1987). Aging furbearers using tooth structure and biomarkers. Wildfurbearer management and conservation in North America.

[15] Grue H, Jensen B (1973). Annular structures in canine tooth cementum in red foxes (Vulpes vulpes L.) of known age.. Dan Rev Game Biol.

[16] Servat A, Feyssaguet M, Blanchard I, Morize JL, Schereffer JL (2007). A quantitative indirect ELISA to monitor the effectiveness of rabies vaccination in domestic and wild carnivores.. J Immunol Methods.

[17] Heaton PR, Johnstone P, McElhinney LM, Cowley R, O'Sullivan E (1997). Heminested PCR assay for detection of six genotypes of rabies and rabies-related viruses.. J Clin Microbiol.

[18] Smith J, McElhinney LM, Heaton PR, Black EM, Lowings JP (2000). Assessment of template quality by the incorporation of an internal control into a RT-PCR for the detection of rabies and rabies-related viruses.. J Virol Methods.

[19] Tamura K, Dudley J, Nei M, Kumar S (2007). MEGA4: Molecular Evolutionary Genetics Analysis (MEGA) software version 4.0.. Molecular Biology and Evolution.

[20] Bourhy H, Kissi B, Audry L, Smreczak M, Sadkowska-Todys M (1999). Ecology and evolution of rabies virus in Europe.. J Gen Virol.

[21] Kuzmin IV, Botvinkin AD, McElhinney LM, Smith JS, Orciari LA (2004). Molecular epidemiology of terrestrial rabies in the former soviet union.. Journal of Wildlife Diseases.

[22] Vanaga S, van der Heide R, Joffe R, van der Poel WH (2003). Rabies in wildlife in Latvia.. Vector Borne Zoonotic Dis.

[23] Anonyme, General EC-HaCD (2008). Report of the task force meeting of the rabies subgroup.. Task force meeting of the rabies subgroup.

[24] Milius J, Razmuviene D, Jacevicius E, Tamošiunas V, Lukauskas K (2004). Epidemiological situation of rabies in Lithuania in 1994–2003.. Vet Zootech.

[25] Maciulskis P, Lukauskas K, Milius J, Jacevicius E, Kiudulas V, Dodet B FA, Muller T, Tordo N (2008). Intake and stability of a rabies vaccine. Towards the elimination of rabies in Eurasia.

[26] Lloyd HG (1980). The red fox.

[27] Kauhala K (1996). Reproductive strategies of the raccoon dog and the red fox in Finland.. Acta Theriologica.

[28] Bruyere V, Vuillaume P, Cliquet F, Aubert M (2000). Oral rabies vaccination of foxes with one or two delayed distributions of SAG2 baits during the spring.. Vet Res.

[29] Cliquet F, Aubert M, Schudel A, Lombard M (2004). Elimination of terrestrial rabies in Western European countries.. Control of infectious animal diseases by vaccination.

[30] Cliquet F, Guiot AL, Munier M, Bailly J, Rupprecht CE (2006). Safety and efficacy of the oral rabies vaccine SAG2 in raccoon dogs.. Vaccine.

[31] Rosatte RC, Lawson KF (2001). Acceptance of baits for delivery of oral rabies vaccine to raccoons.. Journal of Wildlife Diseases.

[32] Cliquet F, Combes B, Barrat J, Dodet B SA, Pastoret PP, Lombard M (2006). Means used for terrestrial rabies elimination in France and policy for rabies surveillance in case of re-emergence.. Rabies in Europe. 2006/08/02 ed.

[33] Robertson CPJ, Baker PJ, Harris S (2000). Ranging behaviour of juvenile red foxes and its implications for management.. Acta Theriologica.

[34] Voigt DR, Tinline RR, Broekhoven LH, Bacon J (1985). A spatial simulation model for rabies control. Population dynamics of rabies wildlife.

[35] Brochier B, Aubert MF, Pastoret PP, Masson E, Schon J (1996). Field use of a vaccinia-rabies recombinant vaccine for the control of sylvatic rabies in Europe and North America.. Rev Sci Tech.

[36] Bachmann P, Bramwell RN, Fraser SJ, Gilmore DA, Johnston DH (1990). Wild carnivore acceptance of baits for delivery of liquid rabies vaccine.. J Wildl Dis.

[37] Knoop EV, Freuling CM, Kliemt J, Selhorst T, Conraths FJ (2010). Evaluation of a commercial rabies ELISA as a replacement for serum neutralization assays as part of the pet travel scheme and oral vaccination campaigns of foxes Berl Münch Tierärztl Wochenschr.

[38] Wasniewski M, Cliquet F (2008).

[39] Zienius D, Zilinskas H, Sajute K, Stankevicius A (2009). Comparative molecular characterisation of the rabies virus in the Lithuanian raccoon dog population.. Bull Vet Inst Pulawy.

[40] Bourhy H, Reynes JM, Dunham EJ, Dacheux L, Larrous F (2008). The origin and phylogeography of dog rabies virus.. J Gen Virol.

[41] Maciulskis P, Lukauskas K, Sederevicius A, Kiudulas V, Pockevicius A (2006). Epidemiology of enzootic rabies in Lithuania.. Medycyna Weterynaryjna.

[42] Masson E, Aubert MF, Barrat J, Vuillaume P (1996). Comparison of the efficacy of the antirabies vaccines used for foxes in France.. Vet Res.

[43] Thulke HH, Selhorst T, Muller T, Wyszomirski T, Muller U (2004). Assessing anti-rabies baiting–what happens on the ground?. BMC Infect Dis.

[44] Anonyme, General EC-HaCD (2009). Report of the task force meeting of the rabies subgroup.. Task force meeting of the rabies subgroup.

[45] Anonyme, General EC-HaCD (2010). Report on the task force meeting of the rabies subgroup.. Task force meeting of the rabies subgroup.

[46] Anonyme (2010). The results of the rabies co-financed eradication programme in the republic of Latwia in 2009..

[47] Smreczak M, Trebas P, Orlowska A, Zmudzinski JF, Dodet B FA, Muller T, Tordo N (2008). Rabies surveillance in Poland (1992–2006). Towards the elimination of rabies in Eurasia.

[48] Aubert MF (1999). Costs and benefits of rabies control in wildlife in France.. Rev Sci Tech.

[49] Freuling C, Selhorst T, Bätza HJ, Muller T, Dodet B FA, Muller T, Tordo N (2008). The financial challenge of keeping a large region rabies-free - the EU example.. Towards the elimination of rabies in Eurasia.

[50] Cliquet F, Barrat J (2008). Rabies. Manual of diagnostic tests and vaccines for terrestrial animals (mammals, birds and bees). 6th ed.

[51] Childs JE, Krebs JW, Real LA, Gordon ER (2007). Animal-based national surveillance for zoonotic disease: quality, limitations, and implications of a model system for monitoring rabies.. Prev Vet Med.

